# Do Electronic Health Records Help or Hinder Medical Education?

**DOI:** 10.1371/journal.pmed.1000069

**Published:** 2009-05-12

**Authors:** Jonathan U. Peled, Oren Sagher, Jay B. Morrow, Alison E. Dobbie

**Affiliations:** 1 Albert Einstein College of Medicine, Bronx, New York, United States of America; 2 Department of Neurosurgery, University of Michigan Health System, Ann Arbor, Michigan, United States of America; 3 Department of Family and Community Medicine, University of Texas Southwestern Medical Center, Dallas, Texas, United States of America

## Abstract

Background to the debate: Many countries worldwide are digitizing patients' medical records. In the United States, the recent economic stimulus package (“the American Recovery and Reinvestment Act of 2009”), signed into law by President Obama, includes $US17 billion in incentives for health providers to switch to electronic health records (EHRs). The package also includes $US2 billion for the development of EHR standards and best-practice guidelines. What impact will the rise of EHRs have upon medical education? This debate examines both the threats and opportunities.

## Abstract 2

Many countries worldwide are digitizing patients' medical records. What impact will these electronic health records have upon medical education? This debate examines the threats and opportunities.

## Jonathan U. Peled and Oren Sagher's Viewpoint: EHRs May Be Hazardous to Medical Education's Health

While the electronic health record (EHR) is a long-overdue innovation in medicine, studies have shown that such records may lead to frustration on the part of health care providers and even harmful outcomes in patients [1],[2]. We also believe that the EHR could have a harmful impact upon medical education.

The first wave of integration of the computer into health care was computerized provider order entry (CPOE)—a computer system that allows direct entry of medical orders by physicians, reducing the risks associated with illegible handwritten orders. CPOE has had mixed effects on medical education [3]–[7]. For example, in a survey of all 143 Johns Hopkins University School of Medicine students who began a Basic Medicine clerkship, 95% of students believed that placing orders helped them learn what tests and treatments patients needed, but students at hospitals that had CPOE faced greater barriers to placing orders themselves than those at hospitals using a paper-based system [3].

CPOE left unaltered the bulk of the teacher–student interaction, since the attending physician still largely relied upon the student to present most of the raw data about the patient. In contrast, the advent of comprehensive EHRs, in which the entire database of patient information (including progress notes, radiographic studies, etc.) is online and available directly to the attending physician, has the potential to fundamentally change the way in which teachers and students interact.

A number of years ago, the clinical settings in which we work went 100% electronic, and since that time we have noticed dramatic changes in the way teachers and students meet and speak together. One of us (JUP) has volunteered at the same student-run free clinic for about six years, during which time he observed the same cohort of physicians teach students for a few years before and a few years after the transition to EHRs. And as a residency program director at an academic medical center, one of us (OS) had the opportunity to witness—from the perspective of the educator—changes in faculty–resident interactions as EHRs were adopted. Since there are only a few published studies on the effects of comprehensive EHRs on medical education [8]–[11], we present here our own observations in the hopes of stimulating further investigation into this important area. We also offer a number of steps that clinical educators can consider to improve the utility of EHRs in medical training.

### EHRs Bypass the Need for Trainees To Synthesize Clinical Information

An important component of medical teaching is the ability to synthesize symptoms, signs, and laboratory results into a coherent story that allows for accurate and efficient medical care—a skill refined by presenting cases. Prior to EHRs, the attending physician was largely dependent upon the trainee for data about the patient, and medical students and residents learned quickly to distill facts and present them in a cogent and effective fashion. Today, in many cases, the attending physician has access to, and will have looked at, the diagnostic studies independently before meeting with the trainee. There is therefore less of an incentive for the student or resident to think critically about blood work or imaging studies beforehand—and EHRs eliminate the need for the trainee to describe these patient data in words. Not only has the attending physician probably seen the chest X-ray already, but the radiologist's dictated report will be available momentarily. These subtle changes can leave the presentation of cases a pro forma educational exercise, rather than the critical moment of intellectual exchange and decision-making.

**Figure 1:**
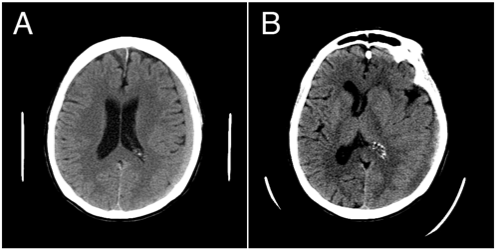
Learning to interpret diagnostic studies may be compromised with the EHR. (A) A 9-mm chronic subdural hematoma causes little in the way of mass effect and does not require surgical intervention. (B) A 9-mm subdural hematoma is causing significant brain shift and is a neurosurgical emergency. The ability to verbally communicate such a difference is an important part of medical training.

Consider computerized tomography studies of two patients presenting with blunt trauma to the head (Figure 1). The resident may describe either finding simply as a “9-mm subdural hematoma.” While this is an accurate description of both scans, the impact of these findings is completely ignored. In the first case (Figure 1A), the hematoma causes little mass effect and can be safely watched, while in the second (Figure 1B), the patient is likely to need an urgent operation to relieve the resultant brain shift. In the presence of EHRs, trainees are guaranteed the option of simply conveying the raw data to the attending physician on the screen in a completely unprocessed manner. While this reduces the likelihood of interpretational error, it also has an insidious effect on the learning process. Transforming patient-specific details into abstract terms (“problem representation”) is a critical part of medical education [12], and if the EHR is not used carefully, it may compromise the development of this important skill.

### EHRs as a Distractor

The architecture of the patient care setting has been completely changed by the ubiquitous presence of computers. In the staff room of the clinic, one now finds rows of young physicians and students lining the walls, staring into computer screens, the silence broken only by the chirping of beepers and the patter of keyboards and double-clicks. In a paper-based clinic, teacher and student often sit next to one another at a conference table to examine documents, allowing those around the table to become engaged. At an EHR clinic where computers line the wall, the two huddle at a monitor, their backs to the room. Few teaching interactions occur spontaneously in such an environment.

Another pitfall arises during the presentation of cases when the need to electronically cosign orders places the teacher at the computer. In the interest of time, some teachers have developed a habit of clicking through the EHR while listening to a trainee present the case. Swiftly and silently, the attending physicians answer their own questions, rather than posing them to their trainees. This practice robs students and residents of the benefits of hearing the questions that the case triggers in the mind of a more experienced clinician.

### Copy and Waste

A recent survey of third-year medical students at the University of Kansas suggested that more teachers take the time to give students feedback on progress notes when they are entered into the EHR [11]. Similarly, many EHR systems allow trainees to send an electronic carbon-copy to their attending physicians on progress notes or other communications, which allows for closer supervision [10]. The value of this carbon-copying cannot be overstated, but such feedback is useful to a trainee only if the trainee actually wrote the note, rather than copying a previous assessment by another provider into today's progress note. Indeed, the pervasiveness of copy and paste within the EHR has been the topic of much debate, since it tends to perpetuate error and inflate volume without corresponding increases in content [13],[14].

### Just in Time Is Not Good Enough

The instant availability not only of progress notes but of all clinical data paradoxically may make it more difficult to keep track of these notes and data. Because residents and students know that data are easily available, they may be less inclined to look them up as the need is not imminent. We have witnessed, for example, a case in which toxic vancomycin levels remained unchecked until rounds when the chief resident specifically inquired about the peak level. The data, it turned out, had been available for 24 hours, but the resident was lulled into not checking since he knew the most current data would be available upon request. He thus robbed himself of the opportunity to learn about managing this clinical problem on his own, and when it was discovered during rounds the senior physicians simply issued orders themselves. All too often, learning opportunities languish in unopened “tabs” or deep within the branches of chart navigation “trees” [15].

### Opportunities for the EHR

Despite the various dangers described above, we acknowledge that EHRs can also offer creative ways to enhance the instruction of medical students and residents. For example, some systems allow users to flag patient records to their own personal “teaching file.” In the future, this feature might be augmented to enable searching for particular words within the records of all patients a user has cared for in the past. One could envision that while speaking with an expert clinician about a certain topic during a didactic session, a trainee might be reminded of a previous patient and be able to immediately access that case. Another advantage is effortless tracking of the number of procedures trainees have performed. Beyond simplifying bookkeeping, some systems allow video recording of entire diagnostic ultrasound sessions for subsequent review, thus extending the bedside teachable moment to dedicated didactic sessions. Perhaps in the not-too-distant future, trainees might be able to record video of bedside procedures from their own eyes' perspective for review and feedback from preceptors.

Far from arguing against the integration of EHRs into clinical practice, we are suggesting that it be done with an awareness of the potential impact on education. There are five steps that might maximize the benefits proffered by the EHR while avoiding some of the pitfalls described above.

First, when appropriate clinically, faculty should actively avoid referring to source data while evaluating clinical presentations by medical students and trainees. Referring to computerized records in advance of, or during such presentations, necessarily robs the trainee of the ability to synthesize information in a cogent fashion. Second, faculty–student and faculty–resident interactions should be fostered by a conducive environment within the outpatient clinic and inpatient ward. One way to accomplish this would be to redesign staff rooms, eliminating the rows of wall-lined computers and reinstating tables in the center of the room that encourage face-to-face interactions. Third, copying and pasting notes could be banned as a matter of policy within training programs, or perhaps even functionally limited within the EHR. Fourth, medical students and residents should be actively encouraged to routinely check laboratory values, rather than relying on the knowledge that results will be available on demand. Finally, it will be important to evaluate the impact of the comprehensive EHR on medical education as more training programs go online through careful research.

### Conclusions

Medical schools and teaching hospitals sit today at an important crossroads. In the United States, the federal government and many third-party payors are demanding that we modernize medical-information systems and have given us an ambitious schedule to do so. Eager to comply with these mandates and anxious to avoid financial penalties, hospitals are implementing EHR systems on a large scale. The effects of this implementation on patient care have not been uniformly positive, and a number of reports of risk have already been published [1],[2]. Our experiences have led us to believe that the potential risk of EHRs to medical teaching may be just as significant and, if not addressed, could erode the education of an entire generation of physicians. On the other hand, if the EHR is used as a *tool* rather than an end unto itself, it will improve our education of young physicians as well as the care of our patients.

## Jay B. Morrow and Alison E. Dobbie's Viewpoint: EHRs Can Enhance Medical Education

EHRs are the future of health delivery in the United States, although their current adoption is far from universal [16]. The potential benefits of EHRs in terms of improved patient care outcomes have yet to be realized. In a 2007 study of 1.8 billion ambulatory visits, use of an EHR resulted in no better outcomes in 14 of 17 health measures [17]. Why have EHRs so far failed to deliver on much of their promise?

The EHR is not a health care delivery *method*; rather, it is a *medium* through which imperfect human providers deliver health care. In and of themselves, EHRs cannot enhance care; their success depends on multiple systems and user-based factors. Many user-based factors are dependent on provider education, and, to date, many practicing providers have received suboptimal training in using the EHR. We contend that learning to conduct patient care within the electronic medium is a complex process that should begin in medical school.

Although US medical schools are increasingly adopting EHRs for patient care and for clinical education, the literature is still sparse on the impact of the electronic record on the medical learner. Many concerns have been raised about potential adverse impacts of the EHR on medical learning. These include the possibilities that: (1) using electronic templates as prompts may reduce students' ability to learn basic history taking and physical exam skills; (2) incorporating the EHR into the encounter may adversely impact communication and threaten the physician–patient relationship; and (3) use of the EHR may adversely impact the clinical teaching and learning environment.

Although research on the impact of EHRs upon medical education is still at an early stage, we believe that, with optimal teaching methods, these three fears will prove unfounded. Indeed, when incorporated efficiently and effectively into the educational environment, we believe that the use of the EHR can enhance medical teaching and learning in three ways.

### The EHR Can Enhance Basic History and Physical Exam Skills

Use of an EHR can enhance history taking and physical exam skills. Our own early research showed that first-year medical students choosing to document a history in the EHR recorded more characteristics of pain than students who chose paper documentation [18]. In our survey of third-year students who had just completed an ambulatory medicine clerkship, 72% reported asking more history questions due to EHR prompts, and 39% ordered more clinical preventive services [11]. Most students (69%) reported that the EHR improved their documentation. In focus groups associated with this study, many students commented that they liked the electronic history prompts, and would “go through the electronic screens in their mind's eye” when deciding on relevant history questions during patient interviews.

### The EHR Can Enhance Physician–Patient Communication

Although we believe that the EHR can enhance physician–patient communication, there is evidence suggesting that physicians may not spontaneously acquire EHR-specific communication skills. These skills include introducing the EHR into the office encounter, adjusting the room's geography to form a physician–patient–computer triad [19], sign-posting to indicate periods of typing or reviewing the EHR for longer than 30 seconds [20], and sharing data with patients on the EHR screen. Emran Rouf and colleagues conducted a cross-sectional survey of patients and physicians to assess the impact of the exam room computer on doctor–patient interactions [21]. Attending physicians and their patients were more comfortable incorporating the EHR into clinical encounters than were residents and their patients. Thus, EHR-specific skills may increase with physician seniority.

We should not, however, wait five to seven years for providers to learn effective EHR communication by trial, error, and experience. Such skills should be taught in medical schools as an integral part of basic communication skills. We have shown that, with four hours of instruction, first-year medical students can demonstrate EHR-specific communication skills from the earliest stage of their clinical education [22]. Outside of the clinical encounter, patients will increasingly demand electronic communication from their providers. A 2006 survey of 1,003 Americans nationwide commissioned by the Markle Foundation, which “works to realize the full potential of information technology to address critical public needs” (http://www.markle.org/), found that 65% of respondents wanted electronic access to their personal health information [23]. Over 90% of respondents expressed that it was important to track their symptoms or health care changes online [23]. As medical educators, it is our duty to train future physicians to address these needs.

### The EHR Can Enhance the Clinical Teaching Environment

Used effectively by skilled teachers, we have found that the EHR can be an impressive clinical teaching tool. Immediate access to clinical data prompts students to practice and demonstrate their clinical reasoning skills in real time. In the so-called RIME (Reporter-Interpreter-Manager-Educator) continuum of medical learning (see, for example, http://www.med.unc.edu/medclerk/grading/rime-framework), used in many US clerkships, information synthesis skills are vital for students to advance to the “Manager” and “Educator” levels [24]. As well as information synthesis, the EHR facilitates just-in-time clinical learning and applied evidence-based medicine at the point of care. Students can access preselected online learning resources and clinical guidelines and apply them immediately to individual patients in clinical settings. The EHR also encourages performance review and facilitates quality improvement, vital skills for tomorrow's practicing physician.

The EHR can promote direct feedback between teacher and student. In our 2007 study, 39% of students reported receiving more feedback on their electronic notes than their paper notes [11]. The flexibility of the EHR allows clinical feedback to be written or verbal, and asynchronous as well as in real time. Students value asynchronous feedback equally to immediate feedback [25].

### Recommendations about Using the EHR To Enhance Clinical Education

In summary, the EHR is not a perfect teaching tool, but it offers several advantages over the paper record. Our recommendations for using the EHR to enhance medical education in clinical settings include:

#### 1. Teach students to document electronically from their earliest clinical experiences

Early instruction allows direct transfer of students' developing skills into the clinical environment, and avoids the added burden of learners needing to assimilate the EHR as they apply their clinical skills in earnest for the first time. Early instruction also allows for suboptimal keyboarding skills to be identified and addressed before the clinical clerkships. Students' keyboarding skills cannot be assumed—in a 2007 focus group of first-year students at our institution, five of eight participants expressed concerns about their keyboard skills. Many students had better texting than typing skills.

#### 2. Emphasize improved communication opportunities

We must train students to communicate synchronously and asynchronously using the EHR. Health care is increasingly delivered outside the traditional hospital admission or doctor's office visit. Better informed patients are demanding asynchronous communication and decision support from their physicians. Medical educators must equip today's graduates to respond to these needs.

#### 3. Conduct faculty development around teaching with the EHR

Many clinical faculty have personally received suboptimal training in using the EHR. Until faculty are expert EHR users, they are unlikely to be expert teachers.

Faculty development on teaching with the EHR should address the geographical layout of computer resources [19], promote comfort with various computer platforms such as laptops and tablets, and retool faculty thinking on the nature and process of the teacher–learner interaction. Faculty who fail to respond to the potential of the EHR within the new clinical teaching environment may suffer the frustrating consequences of limited teaching opportunities, less rich teaching exchanges, and lower-level student responses on the RIME scale [24].

If the EHR is used as a simple repository for patient information, and never as an enhanced communication and quality improvement tool, then it fails to fulfill its potential to improve medical education and patient care.

We believe that if the above recommendations are implemented, then the EHR has the potential to greatly enhance the medical teaching and learning environment, empowering schools to graduate physicians prepared to deliver efficient, effective patient care in the 21st century.

## Peled and Sagher's Response to Morrow and Dobbie

Morrow and Dobbie make a case that with appropriate training of faculty and students, EHRs can enhance medical education. We welcome their recent small pilot study suggesting that teaching medical students EHR-specific communication skills may improve their communications with patients (as assessed using a standardized patient) [22]. We hope that faculty will be as receptive to similar interventions. In Morrow and colleagues' study, the researchers assessed whether or not students organized the clinical setting in a way that allowed the standardized patient to see the computer monitor. This aspect of their study underscores the importance of installing equipment (that is often physically secured and immobile) with an eye toward how it will affect the interactions of the humans who use it.

The EHR is only as good as its user. The pitfalls of EHRs for medical education that we raised above are not meant as an indictment of the medium itself, but rather as a warning about the ways it has been used in these early stages. There is no question that EHRs will shortly supplant the paper chart. We ask only that EHRs be implemented with the necessary forethought about medical education. Such forethought and planning should be oriented toward standardized knowledge metrics and skills assessments, as opposed to surveys of student opinions about their experiences.

There is no doubt that instant access to online learning resources, clinical guidelines, and the primary literature can be a wonderful teaching aide. We agree that an indirect advantage of EHRs for bedside learning is that they force institutions to make the Internet ubiquitously accessible, thus facilitating “just-in-time” learning.

But we disagree that EHR templates improve history and physical exam *skills*. Although they serve as good reminders at the point of care, templates may be associated with two related dangers. First is the ease with which one can allow templated phrases to remain in the record despite not having performed portions of the exam. And second is an assumption by the attending physician that the trainee can adequately perform all of those elements of the examination.

The EHR is an exciting and welcome advance in patient care, and it also stands to enhance the education of the coming generations of physicians. On this, we agree wholeheartedly with Morrow and Dobbie. There is, however, a significant danger in how this exciting new technology is implemented. Its mere presence in the clinic does not make education better or more efficient, and the EHR *implementation strategy* may play a greater role in how successful it is than the technology itself.

## Morrow and Dobbie's Response to Peled and Sagher

We end this collegial debate on the role of EHRs in medical education with consensus on several fundamental points: (1) The EHR holds an inexorable, prime role in the heath care environment of the 21st century; (2) “The EHR is only as good as its user”; (3) Faculty development around EHR education is key; and (4) It is the role of medical education to provide “standardized knowledge metrics and skills assessments.”

All of the above reinforce our call for medical educators to prioritize the integration of EHR skills into medical school curricula.

### We Can Teach Students To Document Electronically from Their Earliest Clinical Experiences

The earlier we introduce EHR skills training into the curriculum, the more they will become a natural part of students' documentation, cognitive processes, clinical perspective, and practice habits.

### The EHR Can Enhance the Clinical Teaching Environment

The EHR provides opportunities to asynchronously reflect on student performance and provide more and better feedback. As they advance through their training, students will encounter EHR documentation features that, without proper training and guidance by attending physicians, would potentially pose patient safety issues.

### We Can Teach Students To Improve Their Communication Efficiency Using the EHR

We mentioned our own research that highlights students' concerns about the effects of the EHR on the physician–patient relationship [11]. The EHR is profoundly changing the current practice environment, yet we are just now in the early stages of rethinking our teaching practices to respond accordingly.

### Next Steps Begin Now

Future studies should evaluate the impact of EHR education on learner outcomes, such as knowledge, skills, and practice behaviors. Evaluating patient-centered outcomes, such as satisfaction, self-efficacy, and self-management, may also underscore the importance of teaching EHR skills to students.

Peled and Sagher have documented the dangers associated with *not* teaching students how to use the EHR to enhance their clinical documentation and communication skills. Early literature shows that the mere presence of the EHR will not improve practice quality [17], and will not make education better or more efficient. As with every other piece of the curriculum, an EHR curriculum has no autopilot setting to produce clinical excellence. We need researchers to lay the curriculum groundwork. And we need motivated medical educators to adeptly integrate EHR skills into their existing curricula to prepare our graduates for 21st century health care practice.

## Abbreviations

CPOE: computerized provider order entry
EHR: electronic health record
